# IGFBP-5 Promotes Fibrosis via Increasing Its Own Expression and That of Other Pro-fibrotic Mediators

**DOI:** 10.3389/fendo.2018.00601

**Published:** 2018-10-15

**Authors:** Xinh-Xinh Nguyen, Lutfiyya Muhammad, Paul J. Nietert, Carol Feghali-Bostwick

**Affiliations:** ^1^Division of Rheumatology and Immunology, Department of Medicine, Medical University of South Carolina, Charleston, SC, United States; ^2^Department of Public Health Sciences, Medical University of South Carolina, Charleston, SC, United States

**Keywords:** fibrosis, insulin-like growth factor binding protein-5 (IGFBP-5), systemic sclerosis (SSc), idiopathic pulmonary fibrosis (IPF), extracellular matrix (ECM)

## Abstract

Pulmonary fibrosis is a hallmark of diseases such as systemic sclerosis (SSc, scleroderma) and idiopathic pulmonary fibrosis (IPF). To date, the therapeutic options for patients with pulmonary fibrosis are limited, and organ transplantation remains the most effective option. Insulin-like growth factor-binding protein 5 (IGFBP-5) is a conserved member of the IGFBP family of proteins that is overexpressed in SSc and IPF. In this study, we demonstrate that both exogenous and adenovirally expressed IGFBP-5 promote fibrosis by increasing the production of extracellular matrix (ECM) genes and the expression of pro-fibrotic genes in primary human lung fibroblasts. IGFBP-5 increased expression of the pro-fibrotic growth factor CTGF and levels of the matrix crosslinking enzyme lysyl oxidase (LOX). Silencing of IGFBP-5 had different effects in lung fibroblasts from normal donors and patients with SSc or IPF. Moreover, we show that IGFBP-5 increases expression of ECM genes, CTGF, and LOX in human lung tissues maintained in organ culture. Together, our data extend our previous findings and demonstrate that IGFBP-5 exerts its pro-fibrotic activity by directly inducing expression of ECM and pro-fibrotic genes. Further, IGFBP-5 promotes its own expression, generating a positive feedback loop. This suggests that IGFBP-5 likely acts in concert with other growth factors to drive fibrosis and tissue remodeling.

## Introduction

Pulmonary fibrosis is a complication of several different diseases such as systemic sclerosis (SSc, scleroderma) and idiopathic pulmonary fibrosis (IPF). SSc is a complex autoimmune disease characterized by progressive fibrosis of the skin and multiple visceral organs (1, 2). Despite active research, the etiology of this connective tissue disease, which causes high morbidity and mortality in the patients, is still unknown. In recent years, SSc-associated lung disease has become the leading cause of death in scleroderma patients (2, 3). Lung fibrosis is also the hallmark of IPF. In fact, IPF and SSc, while being different diseases, show some similarities (4, 5). Pulmonary fibrosis in both of these diseases is characterized by the overproduction of extracellular matrix (ECM) components in the lung. To date, the therapeutic options for patients with pulmonary fibrosis are limited, and lung transplantation remains the most effective treatment (2). Therefore, identifying novel therapeutic targets would significantly advance the treatment of IPF and SSc-associated lung disease.

Insulin-like growth factor-binding proteins (IGFBPs) comprise a family of six secreted proteins that interact with insulin-like growth factors (IGF)-I and II to modulate their bioavailability (6). Although IGFBPs can regulate IGF activity, they also have IGF-independent effects (7). IGFBP-5 is the most conserved member of the IGFBP family and binds IGF-1 with high affinity (7, 8). Similar to other IGFBPs, IGFBP-5 also exerts both IGF-dependent and -independent effects (7, 9, 10).

We previously demonstrated increased expression of IGFBP-5 in skin and lung tissues of patients with SSc and lung tissues of patients with IPF (7, 11, 12). We further showed that IGFBP-5 induced a fibrotic phenotype *in vitro* in primary human pulmonary fibroblasts, *in vivo* in mouse skin and lung, and *ex vivo* in human skin maintained in organ culture (7, 10–15). Furthermore, the expression of IGFBP-5 is increased in bleomycin-induced pulmonary fibrosis in rats (8). Taken together, these findings suggest that IGFBP-5 levels are elevated in the setting of tissue fibrosis and that IGFBP-5 can promote the development of fibrosis.

Multiple growth factors have been implicated in the development and progression of pulmonary fibrosis. Although some of the mechanisms mediating the effects of IGFBP-5 and downstream signaling pathways have been identified, the effect of IGFBP-5 on other growth factors and proteins known to promote fibrosis has not been previously examined. Our goal was to determine whether IGFBP-5 can modulate the levels of known pro-fibrotic factors. Our findings demonstrate that IGFBP-5 increases expression of pro-fibrotic factors, creating a positive feedback loop that may explain how IGFBP-5 triggers fibrosis and perpetuates it.

## Materials and methods

### Lung tissues

Lung tissues were obtained from patients with SSc and IPF undergoing lung transplantation at the Unviersity of Pittsburgh Medical Center under a protocol approved by the Institutional Review Board of the University of Pittsburgh and following written informed consent. Lung tissues were also obtained from organ donors (normal lung; NL) whose lungs were not used for transplantation under a protocol approved by the Institutional Review Board of the University of Pittsburgh.

### *Ex vivo* human lung culture and stimulation

Human normal lung tissues were cut into approximately 3 mm^2^ pieces, and 6 pieces of tissue were placed in each well of a 6-well plate in serum-free Dulbecco's Modified Eagle Medium (DMEM) (Mediatech, Inc., Manassas, VA, USA) supplemented with penicillin, streptomycin, and antimycotic agent (Invitrogen, Carlsbad, CA, USA). Lung tissue cores were treated with 500 ng/ml recombinant IGFBP-5 (rBP5) (GroPep Bioreagents Pty Ltd, Adelaide BC, Australia), a concentration within the physiological range found in the serum of healthy donors (16–19). 10 mM HCl was used as a vehicle control. RNA was extracted from lung tissues after 16 and 30 h of incubation.

### Primary human lung fibroblast culture

Primary human lung fibroblasts were cultured from lung tissues of patients with SSc and IPF undergoing lung transplantation following written consent as previously described (12) under a protocol approved by the University of Pittsburgh Institutional Review Board. Primary fibroblasts were also cultured from the lung tissues of normal donors whose lungs were not used for transplantation (12). Briefly, ~ 2–3 mm^2^ pieces of tissue were minced and fibroblasts were cultured and maintained in DMEM (Mediatech, Inc., Manassas, VA, USA) supplemented with 10% fetal bovine serum (FBS) (Sigma-Aldrich, St. Louis, MO, USA), penicillin, streptomycin, and antimycotic agent (Invitrogen, Carlsbad, CA, USA). Cells were used in passages 2–7.

### *In vitro* fibroblast stimulation

Actively growing primary human lung fibroblasts were stimulated as previously described with some modifications (12). Briefly, 2.0 × 10^5^ primary fibroblasts were plated in 35 mm tissue culture plates in 10% FBS-containing DMEM. After 24 h, the cells were serum-starved in DMEM for 24 h prior to stimulation with 500 ng/ml recombinant IGFBP-5 (GroPep Bioreagents Pty Ltd, Adelaide BC, Australia) or vehicle (10 mM HCl) and harvested after 1 and 3 h for RNA extraction. In addition, primary human lung fibroblasts were infected with adenovirus expressing human IGFBP-5 or a control adenovirus at a multiplicity of infection (MOI) of 50 as we previously described (12).

### Adenovirus construct preparation

The full-length cDNA of human IGFBP-5 was generated as previously described (7, 12), cloned into the shuttle vector pAdlox with a C-terminal triplicate (3x) Flag tag, and used for the generation of replication-deficient adenovirus expressing IGFBP-5-3xFlag in the Vector Core facility at the University of Pittsburgh. Adenovirus expressing 3x Flag tag alone (AdCN-Flag) was used as a control (7, 14).

### Small interfering RNA (siRNA) transfection

Primary human lung fibroblasts were seeded in 35 mm plates 24–48 h prior to transfection with siRNA. Insulin-like growth factor binding protein-5 (IGFBP-5) sequence-specific siRNA and negative control scrambled siRNA were purchased from Dharmacon™ (Lafayette, CO, USA). Transfection was done using Lipofectamine 2000 (Invitrogen, Carlsbad, CA, USA) and 100 nM siRNA diluted in Opti-MEM I Reduced-Serum Medium (Life Technologies, Carlsbad, CA, USA) following the manufacturer's recommendation. Fibroblasts were harvested at 48 h.

### Quantitative PCR

Total RNA was extracted from primary human lung fibroblasts using the RNeasy® mini kit (Qiagen Inc., Valencia, CA, USA). First-strand cDNA was reverse-transcribed with an oligo (dT)12-15 primer (Invitrogen, Carlsbad, CA, USA) and SuperScript IV Reverse Transcriptase (Invitrogen). Gene mRNA expression levels were evaluated by quantitative PCR using the TaqMan® real-time PCR system (Applied Biosystems, Foster City, CA, USA) according to the manufacturer's protocol. Gene expression levels were normalized to glyceraldehyde-3-phosphate dehydrogenase (GAPDH). Relative expression levels of fibroblasts were compared to RNA levels using the comparative CT method formula 2^−ΔΔ*Ct*^. Specific primers and probes for amplifying genes encoding human collagen 1A1 (Col) (Hs00164004_m1), human fibronectin (FN) (Hs00365052_m1), human lysyl oxidase (LOX) (Hs00184700_m1), human IGFBP-5 (Hs00181213_M1), human connective tissue growth factor (CTGF) (Hs01026927_g1), and human GAPDH (Hs02758991_g1) were purchased from Applied Biosystems. Human B2M (Hs00187842_m1) was also used to confirm results obtained with GAPDH with no notable differences (data not shown).

### Western blot analysis

Western blot analysis of fibroblast extracellular matrix fractions was done as previously described (12). The following antibodies were used: fibronectin (FN) monoclonal antibody (clone EP5), collagen type I (COL) polyclonal antibody, GAPDH monoclonal antibody (Santa Cruz, Dallas, TX, USA), vitronectin (VN) polyclonal antibody (Biogenesis, Poole, UK), and horseradish peroxidase-labeled secondary antibody (Santa Cruz, Dallas, TX, USA). Signals were detected using chemiluminescence (ProteinSimple, San Jose, CA, USA).

### Statistical analysis

**Figure 1:**
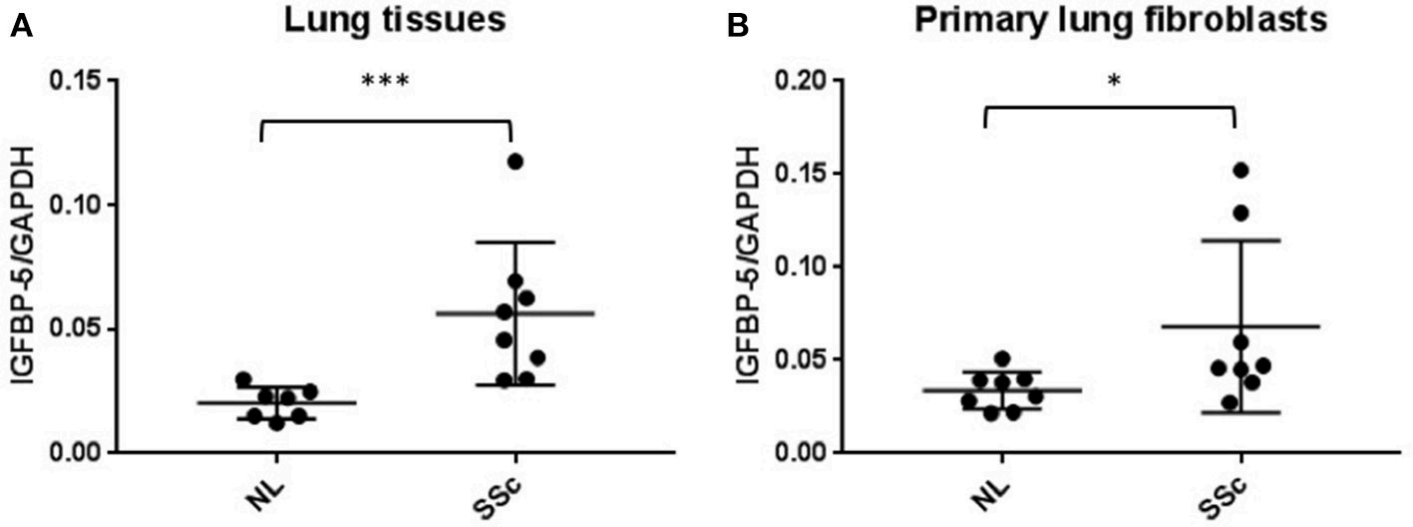
IGFBP-5 mRNA levels are increased in lung tissues and primary fibroblasts of SSc patients. **(A)** Expression of IGFBP-5 was evaluated in lung tissues from 7 normal controls (NL) and 8 patients with SSc pulmonary fibrosis using real-time PCR. **(B)** Expression of IGFBP-5 was evaluated in human primary lung fibroblasts from 8 normal controls (NL) and 8 patients with SSc pulmonary fibrosis using real-time PCR. Each dot represents one individual sample. Graphical presentation of the data analyzed by Mann-Whitney U-test. Values represent mean ± standard deviation. ^*^P < 0.05, ^***^P < 0.001.

For graphical purposes, fold-change estimates were calculated and displayed. For Figure 1, the data was analyzed using the Mann-Whitney U-test with 2-sided *p*-values. For the rest of the figures, due to a lack of normality of the underlying expression level fold-change estimate, comparisons between treatments (i.e., vehicle vs. IGFBP-5, control adenovirus vs. adenovirus expressing IGFBP-5-Flag, scramble siRNA vs. small interfering RNA targeting IGFBP-5) at each time point were conducted using Wilcoxon signed rank tests, which account for the fact that the data are paired within cell lines. Since pro-fibrotic effects of IGFBP-5 have been demonstrated in the past, one-sided hypothesis testing was used for these analyses. *P*-values < 0.05 were considered statistically significant, and no adjustment was made for multiple comparisons. SAS v9.4 (SAS Institute, Cary, NC, USA) or GraphPad Prism version 7 for Windows (GraphPad Software, La Jolla, California, USA) were used for all analyses.

## Results

### IGFBP-5 expression is increased in lung tissues and primary fibroblasts from patients with SSc

We previously reported that IGFBP-5 levels are increased in lung tissues of patients with IPF and primary fibroblasts derived from those lung tissues (12). We also reported that IGFBP-5 expression is increased in dermal fibroblasts from patients with SSc and dermal fibrosis (11). We now show that IGFBP-5 expression is also significantly elevated in lung tissues (Figure 1A) and matching primary fibroblasts (Figure 1B) from patients with SSc-associated lung disease. These and our previous findings suggest that IGFBP-5 is increased in different fibrotic tissues, skin and lung, and across two diseases, IPF and SSc.

### IGFBP-5 induces extracellular matrix and pro-fibrotic gene expression in primary human lung fibroblasts

**Figure 2:**
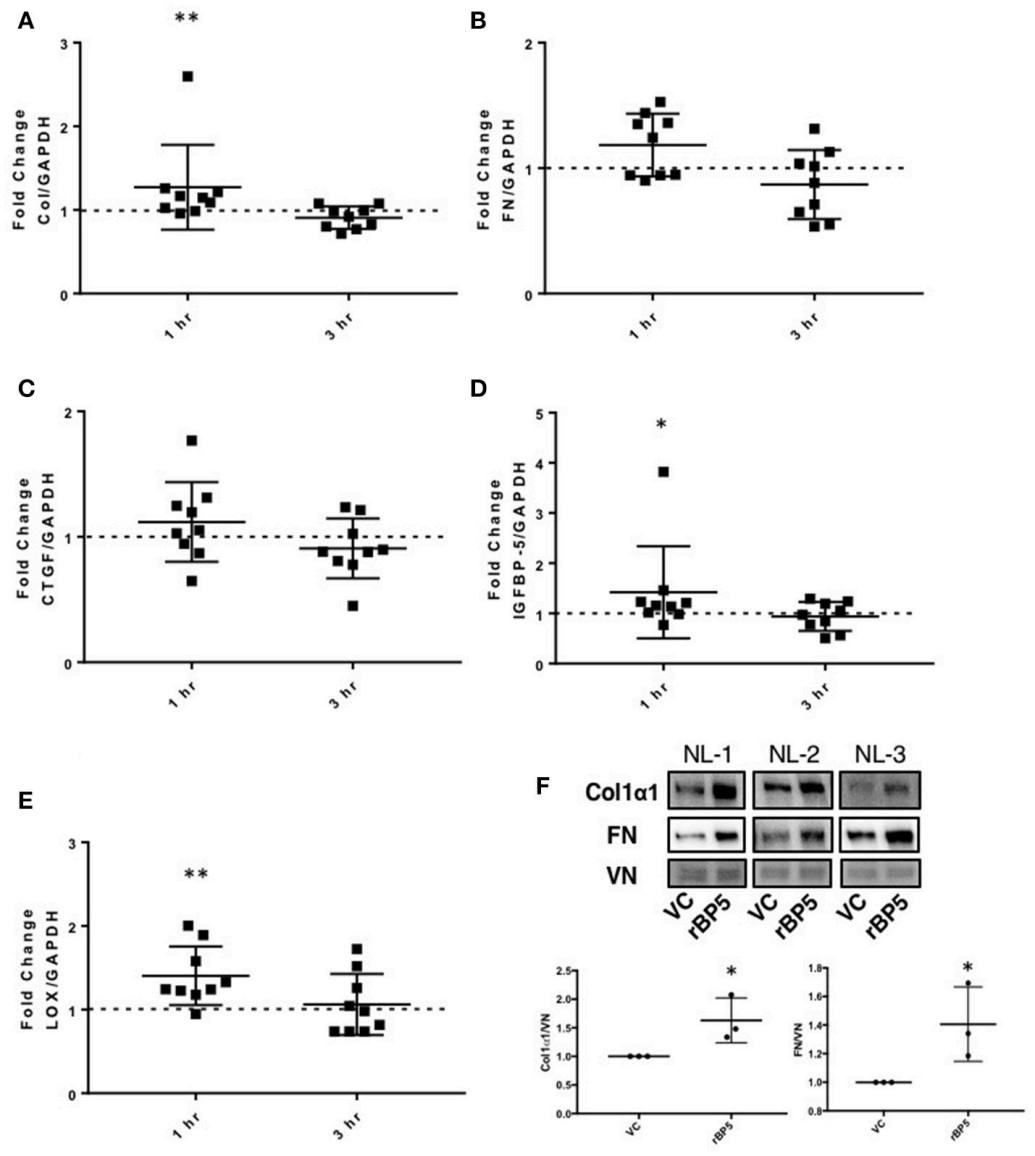
IGFBP-5 exogenously promotes extracellular matrix (ECM) and pro-fibrotic factor production *in vitro*. Primary human lung fibroblasts were treated with vehicle control or recombinant protein IGFBP-5 (rBP5). Samples were harvested after 1 and 3 h of stimulation. Gene expression levels were quantified using qPCR, and fold-change estimates were calculated to compare rBP5 to vehicle. The following genes were analyzed: **(A)** Collagen 1A1. **(B)** FN. **(C)** CTGF. **(D)** IGFBP-5. **(E)** LOX. The data were obtained from 9 different experiments using fibroblasts from lung tissues of 9 different individual normal donors. Graphical presentation of the data analyzed by one-sided Wilcoxon signed rank tests. A dotted line at a fold-change of 1.0 (i.e., which would represent no change) is provided in each graph for reference. **(F)** Immunoblotting results of extracellular matrix fractions of primary human lung fibroblasts from three different donors treated with vehicle or rBP5 for 1 h. Upper images: Collagen1α1 (COL) and Fibronectin (FN) in ECM fractions from an equivalent number of fibroblasts were detected by immunoblotting and signals were normalized to vitronectin (VN). Lower graphs: Graphical presentation of the data analyzed by one-sided paired *t*-test. Values represent mean ± standard deviation. ^*^P < 0.05, ^**^P < 0.01.

We previously showed that IGFBP-5 promotes deposition of collagen and fibronectin in the extracellular matrix fraction of fibroblasts (12, 20). However, we had not examined whether the regulation of ECM production also occurred at the transcriptional level. To determine if exogenous recombinant IGFBP-5 contributes to the development of fibrosis by increasing expression of ECM genes, human primary lung fibroblasts from normal donors were treated with recombinant IGFBP-5 (rBP5) for 1 and 3 h and gene expression was measured using quantitative PCR. rBP5 treatment significantly increased expression of the ECM gene collagen 1A1 (Col) and showed an increasing trend in fibronectin (FN) (Figures 2A,B). Since several pro-fibrotic factors have been implicated in fibrosis, we also examined the effect of IGFBP-5 on CTGF, the ECM-crosslinking enzyme lysyl oxidase (LOX), and IGFBP-5 itself. IGFBP-5 resulted in an increased trend in expression of CTGF (Figure 2C), and significantly increased its own expression (Figure 2D) and the expression of LOX (Figure 2E) within 1 h of stimulation. Expression levels of all genes examined were comparable in IGFBP-5 and vehicle-treated fibroblasts 3 h post-treatment, suggesting that they are immediate early genes downstream of IGFBP-5 and may respond to IGFBP-5 stimulation in a transient manner. The increased production of collagen 1α1 (Col1α1) and fibronectin (FN) was also confirmed at the protein level in fibroblast ECM fractions (Figure 2F and Supplemental Figure 1). Our findings show that increased protein levels can be detected at earlier time points, and the protein response is sustained as we previously reported (10, 12, 20).

**Figure 3:**
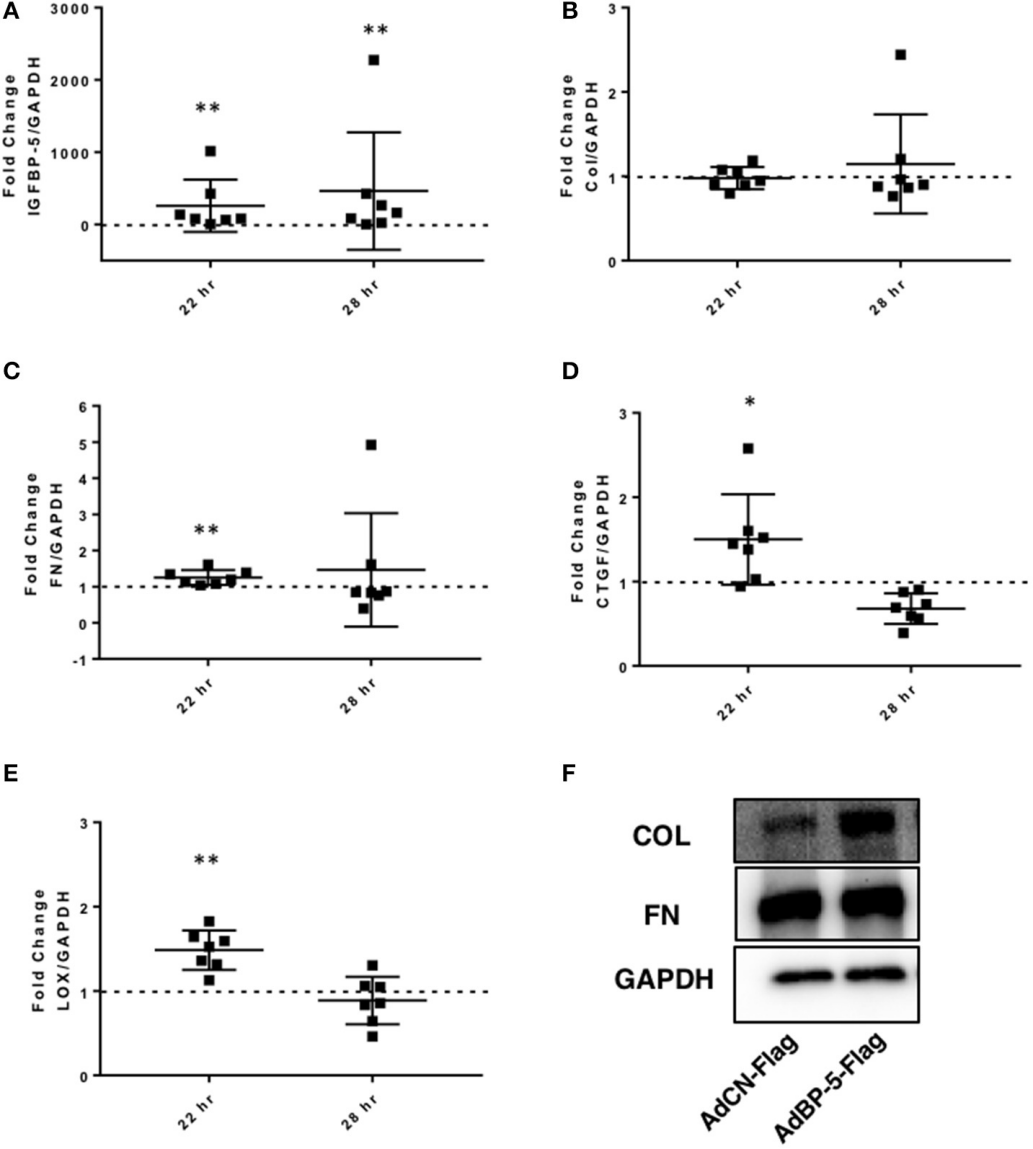
IGFBP-5 expression promotes extracellular matrix (ECM) and pro-fibrotic factor expression *in vitro*. Primary human lung fibroblasts were infected with control adenovirus AdCN-Flag or adenovirus expressing IGFBP-5-Flag (AdBP-5-Flag). Samples were harvested after 22 and 28 h of infection. Levels of expression were quantified using qPCR at 22 and 28 h, and post-infection fold-change estimates were calculated to compare AdBP-5-Flag to control. The following genes were analyzed: **(A)** IGFBP-5. **(B)** Collagen 1A1. **(C)** FN. **(D)** CTGF. **(E)** LOX. The data are obtained from 7 different experiments using fibroblasts from lung tissues of 7 different normal donors. Graphical presentation of the data analyzed by one-sided Wilcoxon signed rank tests. Values represent mean ± standard deviation. A dotted line at a fold-change of 1.0 (i.e., which would represent no change) is provided in each graph for reference. **(F)** Representative protein levels of Collagen 1A1 (Col) and Fibronectin (FN) in the ECM fraction from an equivalent number of fibroblasts infected with AdCN-Flag or adenovirus expressing IGFBP-5-Flag (AdBP-5-Flag) for 24 hr and analyzed by immunoblotting. GAPDH was detected in the corresponding lysates using immunoblotting. ^*^P < 0.05, ^**^P < 0.01.

To further confirm the ECM-promoting effect of exogenous IGFBP-5 and compare it to adenovirally-mediated overexpression of the protein, IGFBP-5 was expressed in normal human primary lung fibroblasts using replication-deficient adenovirus as we previously described (7, 12). Primary lung fibroblasts were infected with adenovirus expressing full length human IGFBP-5 (AdIGFBP-5-3xFlag) or a control adenovirus (AdCN-3xFlag) for 16 h after which the media was changed and incubation was continued for an additional 6 or 12 h, corresponding to 22 and 28 h of total infection time, respectively. These time points were selected as they represent the earliest time points when adenovirally encoded IGFBP-5 protein is detected in the supernatants of primary lung fibroblasts (data not shown). We first confirmed expression of human IGFBP-5 in fibroblasts infected with AdIGFBP-5-3xFlag. Significantly increased IGFBP-5 expression levels were noted at both 22 and 28 h (Figure 3A). Adenovirally-mediated expression of IGFBP-5 was on average 259- and 463-fold higher than control virus-infected cells at 22 and 28 h, respectively. Collagen 1A1 expression showed a trend toward an increase at 28 h (Figure 3B), although the difference did not reach statistical significance, unlike the response to rBP5 treatment. IGFBP-5 expression significantly increased mRNA levels of FN, CTGF, and LOX at 22 h (Figures 3C–E). Although AdIGFBP-5 infection significantly increased levels of CTGF at 22 h, it reduced its expression levels 28 h post-infection. Thus, both exogenously added recombinant IGFBP-5 and adenovirally expressed IGFBP-5 induce the expression of ECM and pro-fibrotic factors in primary human lung fibroblasts. We further validated increased ECM protein levels in the extracellular matrix fraction of fibroblasts using a representative donor fibroblast strain that had shown increased ECM gene transcription in response to IGFBP-5 (Figure 3F). Interestingly, increased collagen 1α1 (Col1α1) was noted in the ECM fraction of fibroblasts expressing IGFBP-5 for 24 h, although mRNA levels were not significantly increased. This could in part be due to the fact that IGFBP-5 acts by protecting ECM protein from degradation (20). The increased deposition of ECM proteins in the matrix of fibroblasts confirms our previously reported findings (12).

### Silencing of IGFBP-5 has different effects on the expression of extracellular matrix and pro-fibrotic genes in fibroblasts from normal and fibrotic lung tissues

**Figure 4:**
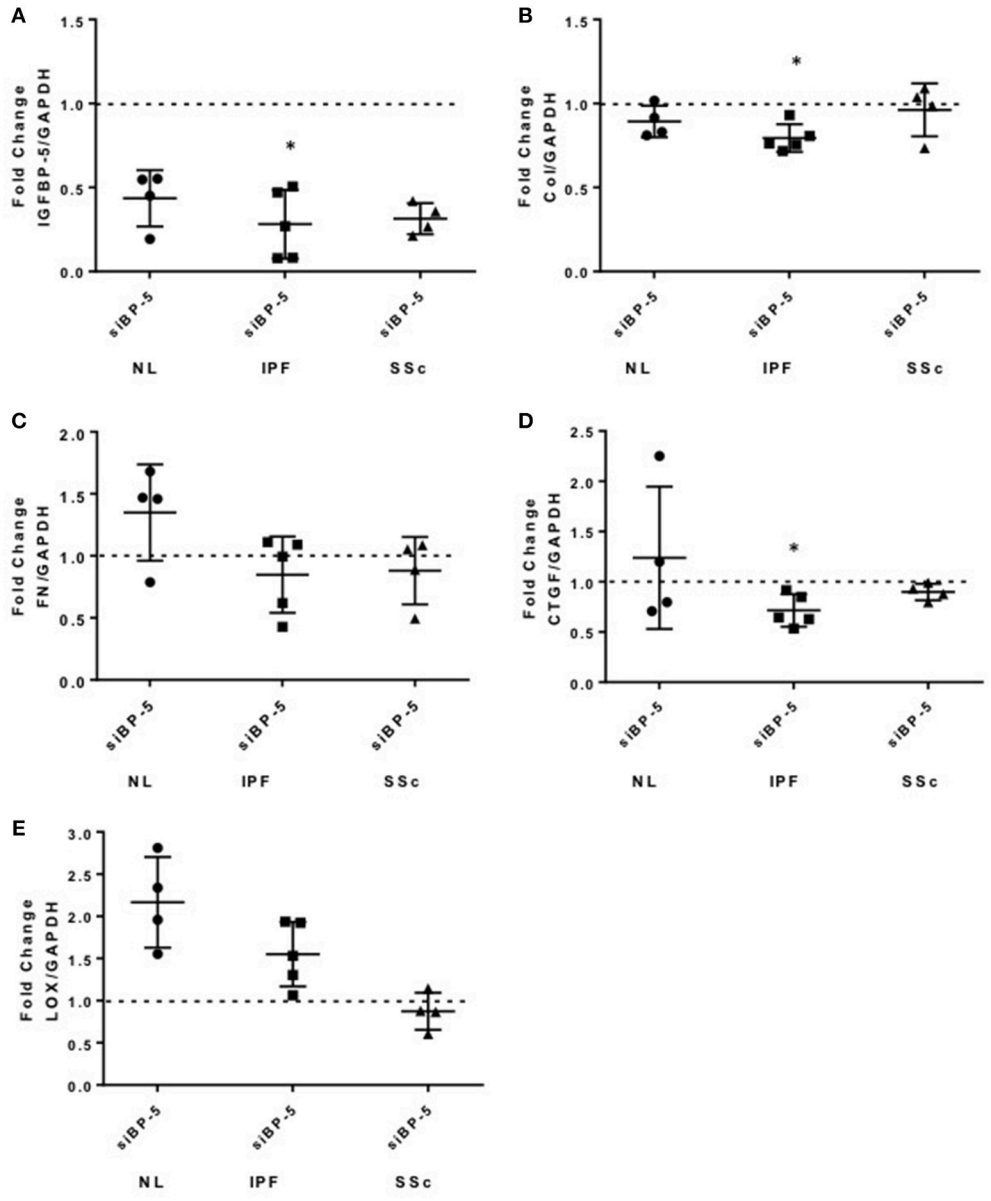
Knockdown of IGFBP-5 shows variable effects in primary pulmonary fibroblasts from different donors. IGFBP-5 was silenced in primary human lung fibroblasts from normal lung (NL, circles) and lung tissues of patients with IPF (squares) and SSc (triangles) using small interfering IGFBP-5 (siBP-5) or scramble siRNA as a negative control (siCN) for 48 h. Levels of expression were quantified using qPCR, and fold-change estimates were calculated to compare siBP-5 to siCN. The following genes were analyzed: **(A)** IGFBP-5. **(B)** Collagen 1A1. **(C)** FN. **(D)** CTGF. **(E)** LOX. The data shown are obtained using fibroblasts from 4 different normal donors (NL), 5 patients with IPF and 4 patients with SSc. Graphical presentation of the data analyzed by one-sided Wilcoxon signed rank tests. A dotted line at a fold-change of 1.0 (i.e., which would represent no change) is provided in each graph for reference. Values represent mean ± standard deviation. ^*^P < 0.05.

We previously reported increased IGFBP-5 production by fibroblasts from fibrotic skin and lung tissues (11, 12). To further understand the role of IGFBP-5 in the development of fibrosis, we silenced endogenous IGFBP-5 in primary human lung fibroblasts derived from lung tissues of normal donors (NL) and patients with IPF or SSc using a sequence-specific siRNA (siBP-5). Scrambled siRNA that in our experience parallels gene expression levels in untreated fibroblasts was used as a control (siCN). We first confirmed efficient silencing of IGFBP-5 expression using siBP5 (Figure 4A). siBP-5 resulted on average in 57, 72, and 69% reduction in IGFBP-5 mRNA levels in NL, IPF, and SSc primary human lung fibroblasts, respectively. Decreased IGFBP-5 expression was significant in IPF fibroblasts (*P* = 0.03) and trended toward significance in NL and SSc fibroblasts (*P* = 0.06). As shown in Figure 4B, silencing of IGFBP-5 in IPF fibroblasts significantly reduced Col1A1 expression, but had no significant effects on the expression levels of FN (Figure 4C). For the pro-fibrotic factors, silencing IGFBP-5 reduced CTGF levels in IPF fibroblasts (Figure 4D), whereas silencing endogenous IGFBP-5 increased LOX expression (Figure 4E) at the examined time point of 48 h. Similar to what we observed in IPF fibroblasts, silencing endogenous IGFBP-5 in NL fibroblasts also increased LOX expression (Figure 4E). Increases in LOX expression did not reach statistical significance as our hypothesis was one-sided. In NL and SSc fibroblasts, silencing IGFBP-5 had no effect on the other genes examined, although CTGF levels showed a trend toward decrease in SSc fibroblasts (*P* = 0.06). Thus, the effect of silencing endogenous IGFBP-5 in healthy and diseased lung tissue fibroblasts had different effects on the expression of ECM and pro-fibrotic genes. The difference in response of cells from different donors and diseases may be due to the different extent of silencing of IGFBP-5, with more efficient silencing of endogenous IGFBP-5 noted in IPF fibroblasts.

### IGFBP-5 induces expression of ECM and pro-fibrotic factors *ex vivo*

**Figure 5:**
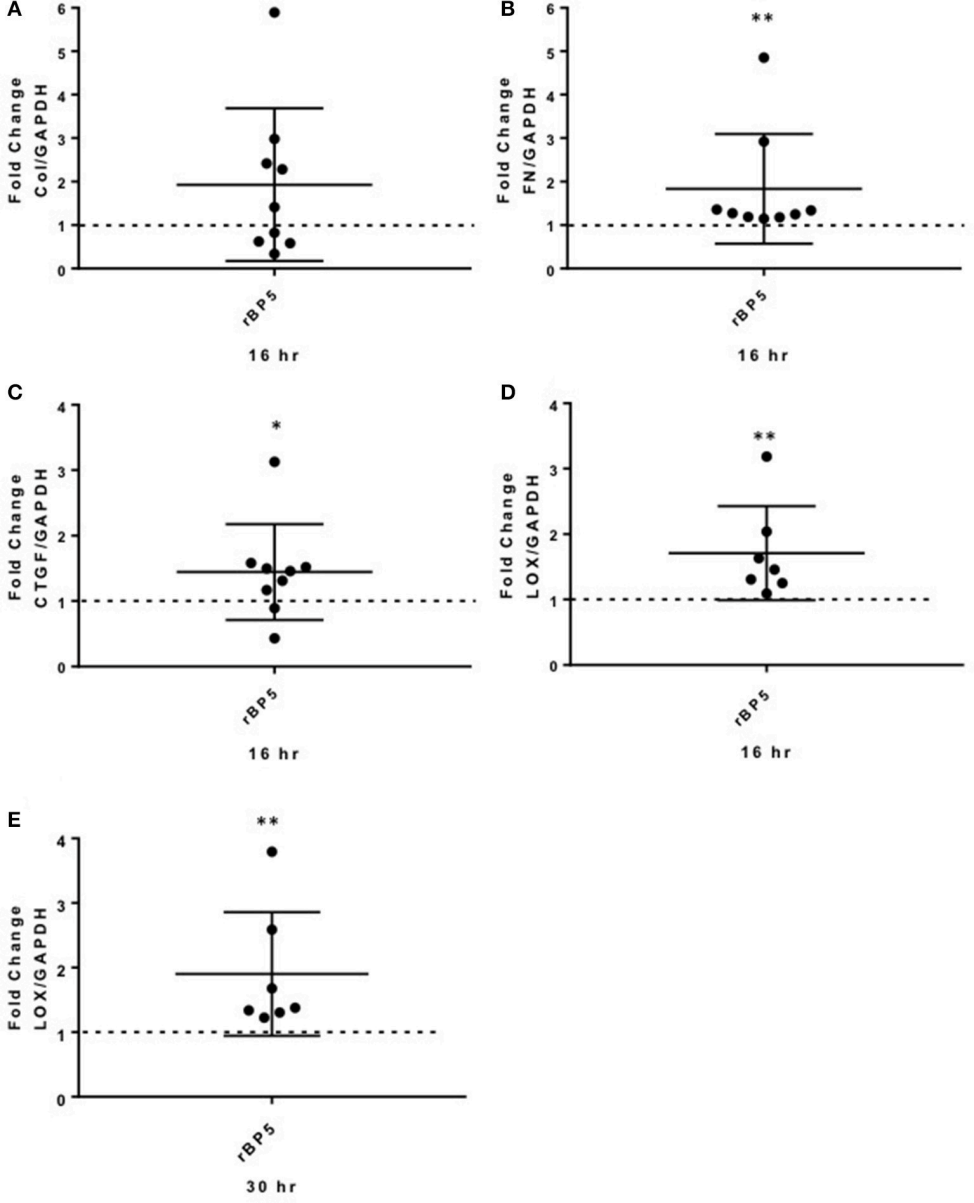
IGFBP-5 promotes ECM and pro-fibrotic factor production *ex vivo* in human lung tissues. Human lung tissue cores were treated with vehicle control (VC, 10 mM HCl) or recombinant IGFBP-5 (rBP5, 500 ng/ml) for 16 and 30 h. Gene expression levels were measured using qPCR at the indicated time points, and fold-change estimates were calculated to compare IGFBP-5 to VC. The following genes were analyzed: **(A)** Collagen 1A1 mRNA levels at 16 h. **(B)** FN mRNA levels at 16 h. **(C)** CTGF mRNA levels at 16 h. **(D)**. LOX mRNA levels at 16 h. **(E)** LOX mRNA levels at 30 h. The data shown are obtained from lung tissues of 7–9 different donors. Graphical presentation of the data analyzed by one-sided Wilcoxon signed rank tests. A dotted line at a fold-change of 1.0 (i.e., which would represent no change) is provided in each graph for reference. Values represent mean ± standard deviation. ^*^P < 0.05; ^**^P < 0.01.

To extend our findings from the *in vitro* studies using primary fibroblasts, we examined the effect of rBP5 *ex vivo* in human normal donor lung tissues maintained in organ culture. Lung tissue cores were treated with rBP5 (500 ng/ml) or 10 mM HCl as a vehicle control for 16 and 30 h. Recombinant IGFBP-5 increased expression of the ECM genes Col1A1 and FN (Figures 5A,B), although the increase in Col1A1 did not reach statistical significance whereas that of FN did. IGFBP-5 also significantly increased expression of CTGF in human lung tissues (Figure 5C). Furthermore, IGFBP-5 significantly increased LOX expression (Figures 5D,E). Although the increase in ECM and CTGF occurred at 16 h and was reduced by 30 h (data not shown), the induction of LOX was sustained through 30 h. This suggests that the increase in ECM occurs early while the increase in the levels of LOX is sustained, thus providing longer ECM crosslinking activity. Thus, IGFBP-5 can promote expression of ECM and fibrotic genes *ex vivo* in human lung tissues, validating and extending our *in vitro* findings.

## Discussion

Pulmonary fibrosis is a manifestation of diseases such as SSc and IPF. Elevated levels of IGFBP-5 have been detected in primary fibroblasts from both of those diseases and *in vivo* in fibrotic lung tissues (12). Further, IGFBP-5 expression is increased in liver fibrosis *in vivo* and during hepatic stellate cell (HSC) transdifferentiation *in vitro* (21, 22). IGFBP-5 has also been implicated in different types of cancers such as breast, ovarian, and colorectal cancer as well as in wound healing and tissue regeneration (6, 23–26). The effects of IGFBP-5 are known to be cell type- and tissue-specific.

Fibroblasts are essential effector cells responsible for the increased production of ECM and thus fibrosis in different organs (27). As a result, examining the response of fibroblasts to both pro- and anti-fibrotic factors is essential for elucidating mechanisms that regulate these cells in the setting of fibrosis. In fact, fibroblasts have been widely used for assessing the effect of pro-fibrotic factors and for testing the efficacy of potential anti-fibrotic therapies (27). To complement findings using pulmonary fibroblasts, lung tissue slices have been used to extend *in vitro* findings and establish their relevance in a human tissue. We have used a similar approach with our own modifications using lung tissue cores rather than slices for assessing the effects of IGFBP-5 in human lung tissues. This allows us to extend our findings from isolated cells in culture and use a model that is more comparable to the *in vivo* milieu.

In this study, we investigated the effect of endogenous and exogenous IGFBP-5 on fibroblast production of ECM components and factors involved in the promotion of fibrosis in different organs. Our results demonstrate that the increase in IGFBP-5 levels is a primary and early event in pulmonary fibrosis since IGFBP-5 induces expression of ECM and pro-fibrotic genes as early as 1 h post-stimulation. The rationale for examining both exogenously added recombinant IGFBP-5 and the adenovirally expressed form is that these forms of IGFBPs may exert different effects (28, 29). As we previously reported (12), both adenovirally-expressed and exogenous IGFBP-5 promote ECM deposition in primary human lung fibroblasts (12). In contrast, in osteosarcoma cells, endogenous and exogenous IGFBP-5 have been shown to exert opposite effects (29). Further, Yamaguchi et al. (20) demonstrated a role for IGFBP-5 trafficking into fibroblasts and ECM-protective effects of extracellular IGFBP-5 (20). Our findings suggest that endogenous and exogenous IGFBP-5 may exert similar effects on the expression of certain genes such as ECM components and CTGF which were induced by rBP5 and reduced by silencing endogenous IGFBP-5. In contrast, our data show that endogenous and exogenous IGFBP-5 may exert opposite effects on other genes examined in the same cells. This is the case for LOX expression which was induced by recombinant IGFBP-5 and silencing of endogenous IGFBP-5. It is possible that endogenous IGFBP-5 may provide an inhibitory effect and silencing endogenous expression would support the pro-fibrotic effects of recombinant exogenous IGFBP-5. Thus, understanding the effect of both endogenous and exogenous IGFBP-5 will lead to a better understanding of the role that compartmentalization of this protein plays in fibrosis. Our findings support the concept that endogenous and exogenous IGFBP-5 might exert different effects in primary human lung fibroblasts from healthy and diseased tissues as well. Since IGFBP-5 is a secreted protein, this suggests that localization of IGFBP-5 intracellularly or extracellularly may dictate its effects on cell function.

We had previously shown that IGFBP-5 can trigger a fibrotic phenotype *in vitro* in primary human fibroblasts, *in vivo* in mouse skin and lung, and *ex vivo* in human skin in organ culture (12–15). However, the question as to whether the promotion of fibrosis was directly mediated by IGFBP-5 or via other pro-fibrotic factors that may be downstream of IGFBP-5 had remained unanswered. In fact, our data show that IGFBP-5 not only directly induced the expression of ECM genes such as collagen I and fibronectin, but it also increased the expression of pro-fibrotic genes such as CTGF and IGFBP-5 itself. Further, IGFBP-5 increased the expression of LOX, an enzyme responsible for the covalent crosslinking of extracellular matrix proteins such as collagen and elastin (30, 31). Elevated expression of cytokines and growth factors with pro-fibrotic activity such as CTGF have been reported in SSc and related diseases. For example, the pro-fibrotic activity of CTGF is well documented. Mori et al. (32) reported that subcutaneous injection of TGF-β and CTGF promoted dermal fibrosis. The investigators demonstrated that persistent fibrosis required both CTGF and TGF-β stimulation and that CTGF alone caused little granulation (32). In contrast, others have shown that CTGF is required for bleomycin-induced skin fibrosis in mice (33), and transgenic targeted expression of CTGF alone in fibroblasts promotes fibrosis in different organs including skin, lung, kidney, and vasculature (34). The critical role of CTGF in fibrosis has been the focus of ongoing research and the development of potential therapies targeting this growth factor (35). For example, a recent study by Makino et al. (36) examined the therapeutic effect of CTGF inhibition using anti-CTGF monoclonal antibody (36). CTGF inhibition reduced inflammation and vascular damage in a murine model of fibrosis (36). We propose that IGFBP-5 promotes pulmonary fibrosis by directly inducing expression of ECM genes and by increasing levels of other pro-fibrotic proteins such as CTGF, resulting in further increase of ECM production. Thus, IGFBP-5 is likely to promote fibrosis by working in concert with other pro-fibrotic factors, such as CTGF, which then potentiate the anti-fibrotic activity of IGFBP-5.

In addition to regulating ECM and growth factor genes, IGFBP-5 also increased expression of LOX, an enzyme responsible for cross-linking the matrix. Thus, in addition to increasing expression of ECM components, IGFBP-5 also promotes their cross-linking via increasing LOX levels, thus altering tissue structure and function. The critical role of LOX in fibrosis has been demonstrated in several studies. For example, targeting LOX has been shown to reduce peritoneal fibrosis (37) cardiac fibrosis (38) and pulmonary fibrosis (39–41). LOX has even been proposed as a biomarker of fibrosis in patients with SSc (42). Thus, induction of LOX by IGFBP-5 further potentiates the pro-fibrotic effects of IGFBP-5 by increasing the enzymatic crosslinking of collagen and other matrix components, rendering the ECM more resistant to proteolytic degradation.

The induction of pro-fibrotic genes by IGFBP-5 showed an immediate early response pattern. Such a pattern has been reported for other pro-fibrotic factors such as TGF-β which increases the expression of genes such as early growth response (Egr-1) (43), inhibitor of differentiation 1 (Id1) (44, 45) and CTGF (46) early while it induces collagen levels in a delayed manner. Growth factors typically induce an immediate early transcriptional response that is independent of protein synthesis and a more delayed response that requires proteins. Often, these immediate early genes are transcription factors. The immediate early response to IGFBP-5 stimulation of fibroblasts follows the pattern typical of growth factors in the kinetics of its effects, although its immediate early gene response includes ECM components and growth factors. We recently showed that IGFBP-5 with a mutated NLS that abrogates its nuclear localization retains the ability to induce dermal fibrosis when expressed in primary human fibroblasts or in human skin in organ culture using an adenoviral vector (7). This may reflect (a) the ability of secreted IGFBP-5 to bind ECM components and protect them from degradation, thus promoting ECM accumulation and fibrosis, and/or (b) the possibility that cytoplasmic IGFBP-5 can promote the translocation of a “partner” factor/protein to the nucleus, thus exerting transactivating effects, which might be further facilitated by target genes being in a “transcriptionally poised” state—a chromatin state that allows for rapid gene activation—thus allowing for an “immediate early” gene response.

Growth factors mediating their effects via other cytokines or pro-fibrotic factors has also been previously noted in the scientific literature. For example, polypeptide growth factors such as the platelet-derived growth factor (PDGF) (47) family and the epidermal growth factor (EGF) (48) family are also known to mediate pro-fibrotic effects by inducing expression of extracellular matrix genes directly or as mediators of the effects of TGF-β (47, 48). Further, the pro-fibrotic factor, IL-6 is known to induce collagen directly, and to also regulate IGFBP-5 (49, 50). O'Reilly et al. (50) examined the effects of IL-6 *trans* signaling in mediating fibrosis. Their study showed that IL-6 mediated fibrosis through enhanced TGF-β signaling which was due to Gremlin-1, a bone morphogenetic protein antagonist and a member of TGF-β family (50). We now add IGFBP-5 as an upstream regulator of pro-fibrotic growth factor gene expression, suggesting that IGFBP-5 regulation of genes such as CTGF may be one of the mechanisms that sustain its fibrotic effects.

To further delineate the role of endogenous IGFBP-5 in primary fibroblasts, we silenced IGFBP-5 expression in fibroblasts from normal donors and patients with IPF and SSc. Knock down of IGFBP-5 showed that NL, IPF, and SSc fibroblasts respond differently to a reduction in endogenous IGFBP-5 expression. Since silencing does not result in a complete loss of IGFBP-5, it is plausible that the variable effects are due to the fact that fibroblasts from different diseases and different individuals vary in their sensitivity to IGFBP-5 and that some retain their phenotype with residual low levels of expression of IGFBP-5. Use of tissue-derived primary fibroblasts from different donors has inherent challenges with respect to variability of respone to stimuli (which is noted even in response to potent pro-fibrotic factors such as TGF-β), however it provides greater relevance of findings to human disease than immortalized cell lines. Several factors may contribute to the variability of the response, including different kinetics in fibroblasts from different donors, variable levels of the IGFBP-5 “receptor,” dissimilar levels of secreted proteases that target IGFBP-5, as well as differing propensities of individuals to develop fibrosis, to name a few. In contrast to the variability we see with primary fibroblasts, IGFBP-5 silencing was shown to consistently affect the survival of hepatic stellate cells in liver fibrosis due to increased cell apoptosis (22). Our findings show that reducing endogenously expressed IGFBP-5 does not necessarily impair the fibrotic phenotype in all fibroblasts examined, at least within the duration of transient *in vitro* silencing and to the extent of silencing observed in fibroblasts from different donors, and confirm the diverse functions of IGFBP-5 in different cells. They further suggest that targeting extracellular IGFBP-5 in diseases such as SSc or IPF where IGFBP-5 levels are elevated may be a more appropriate strategy for ameliorating fibrosis.

In summary, our data builds on our previous findings and provides new compelling evidence that IGFBP-5 is directly involved in the pathogenesis of pulmonary fibrosis by increasing production of extracellular matrix proteins and indirectly by inducing expression of growth factors that promote and sustain fibrosis. IGFBP-5 also functions in an autocrine manner to increase its own expression, further potentiating the fibrotic effect. Our current study establishes the role of IGFBP-5 as an important mediator in fibrosis that is upstream of known pro-fibrotic factors, suggesting that strategies to inhibit IGFBP-5 function might be effective for the amelioration of fibrosis.

## Ethics statement

This study was carried out in accordance with the recommendations of the Medical University of South Carolina and University of Pittsburgh Institutional Review Boards (IRB) with written informed consent from all subjects. All subjects gave written informed consent in accordance with the Declaration of Helsinki. The protocol was approved by the IRB of our institution where tissues were obtained.

## Author contributions

X-XN performed the research, collected data, analyzed and interpreted data, and wrote the manuscript. LM and PN analyzed the data. CF-B designed the research, interpreted data, supervised and organized the study, wrote and edited the manuscript.

### Conflict of interest statement

The authors declare that the research was conducted in the absence of any commercial or financial relationships that could be construed as a potential conflict of interest.

## Supplementary material

Supplemental Figure 1 Original uncropped images for Figure 2F showing that IGFBP-5 increases collagen and fibronectin in the ECM fraction.
