# Supplementary figures and images for: IGFBP-5 Promotes Fibrosis via Increasing Its Own Expression and That of Other Pro-fibrotic Mediators
Source: Front Endocrinol (Lausanne). 2018 Oct 15;9:601. doi: 10.3389/fendo.2018.00601 (PMC6196226; doi:10.3389/fendo.2018.00601)

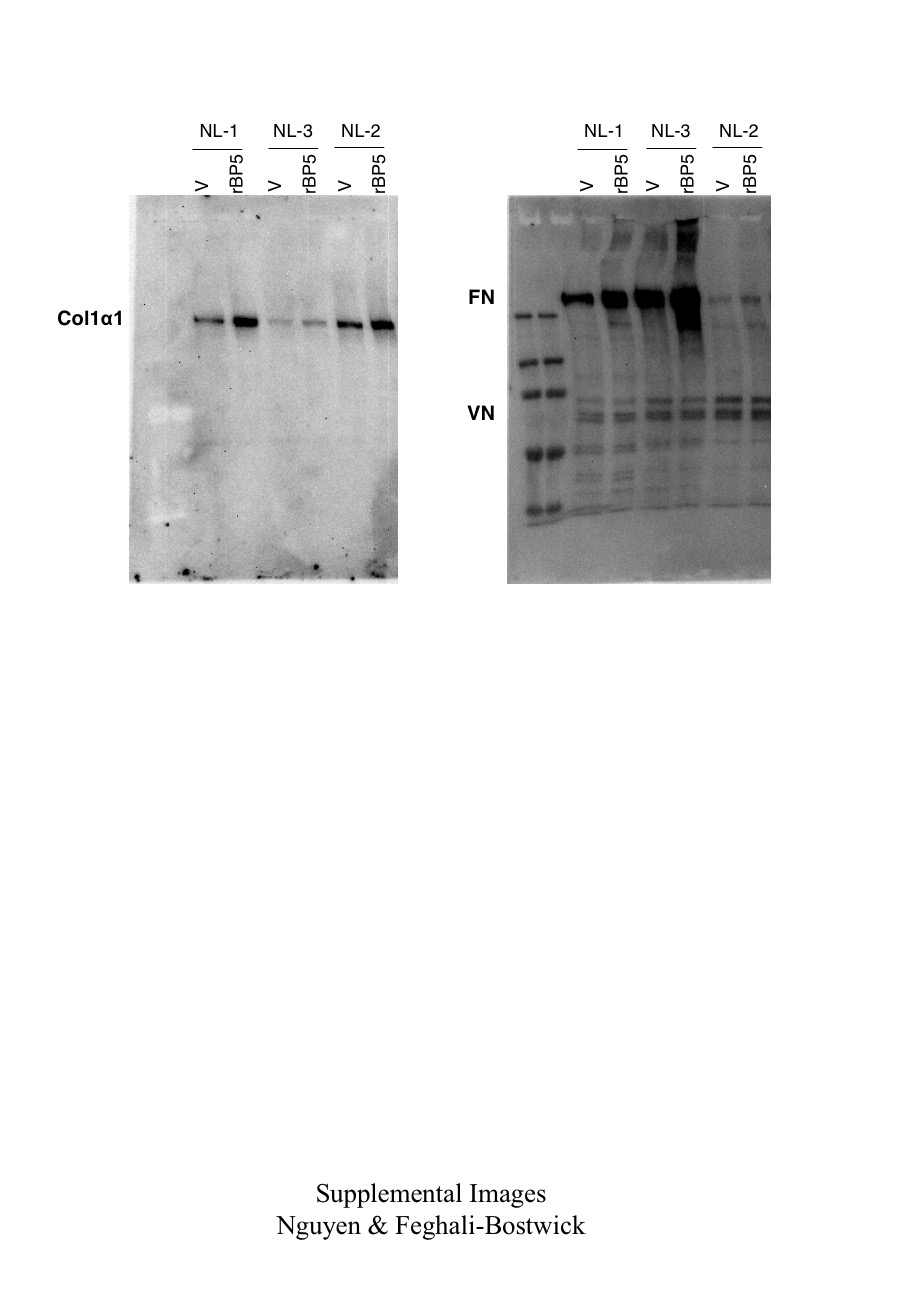

Supplement: Supplemental Figure 1 — Original uncropped images for Figure 2F showing that IGFBP-5 increases collagen and fibronectin in the ECM fraction. [file Image_1.JPEG]
